# Multidisciplinary recommendations for routine follow-up of isocitrate dehydrogenase‑mutant glioma in 2026: Adapting clinical practice to the molecular era

**DOI:** 10.1093/nop/npag032

**Published:** 2026-04-10

**Authors:** Mary Jane Lim-Fat, Amelie Darlix, Bogdana Suchorska, Antonella Castellano, Giuseppe Minniti, Maarten Wijnenga, Susan C Short, Martin J van den Bent

**Affiliations:** Division of Neurology, Department of Medicine, Sunnybrook Health Sciences Centre, Toronto, Canada; Department of Medical Oncology, Institut Régional du Cancer de Montpellier, University of Montpellier, Montpellier, France; Institute of Functional Genomics, University of Montpellier, CNRS, INSERM, Montpellier, France; Department of Neurosurgery, Heidelberg University Hospital, Heidelberg, Germany; Department of Radiotherapy, Vita-Salute San Raffaele University, Milan, Italy; Neuroradiology Unit and CERMAC, IRCCS Ospedale San Raffaele, Milan, Italy; Department of Radiological Sciences, Oncology and Anatomical Pathology, Sapienza University of Rome, Rome, Italy; IRCCS Neuromed, Pozzilli, Italy; Brain Tumor Center at Erasmus MC Cancer Institute, Rotterdam, the Netherlands; Department of Radiotherapy, Leeds Institute of Medical Research, St James’s Hospital, Leeds, UK; Brain Tumor Center at Erasmus MC Cancer Institute, Rotterdam, the Netherlands

## Abstract

Grade 2 and most grade 3 isocitrate dehydrogenase (IDH) mutant diffuse gliomas are slow growing tumors, for which however no curative treatment is available. Although surgery as extensive as possible improves outcome, for virtually all patients at some point in time further treatment is necessary. Treatment with radiotherapy and chemotherapy is very effective in controlling tumor growth in most patients, but has side effects. Therefore, in many patients these are postponed and a postoperative “watch-and-wait” observational strategy is followed. With the approval of IDH inhibitors, another effective strategy has emerged allowing further delay of treatment with radiotherapy and chemotherapy. This underscores the need for guidance how to best follow these patients during their subsequent phases of treatment, but most guidelines give only minimal recommendations for follow-up. Follow-up should not be limited to imaging only, but should also routinely assess seizures and cognition of patients. Follow-up intervals should be based on the risk of progression, allowing longer intervals for patients with oligodendroglioma, for more definitively treated patients and for patients with longer lasting disease stabilization. Evidence justifying a more intensive follow-up of patients on IDH inhibitors than for patients on a “watch-and-wait” strategy is lacking. Assessing a growth trajectory over time and routine two-dimensional or three-dimensional tumor assessment will allow identification of early changes in growth rate. This manuscript provides guidance for optimal follow-up of these patients with perspectives from all involved disciplines, as indeed the follow-up of these patients needs to be truly multidisciplinary, at every step along the pathway of these patients.

Key Points

- The standardized MR imaging protocol should be routinely used for patients with an IDH-mutant glioma.
- The follow-up intervals should be individualized and based on the risk of progression.
- Assessing growth trajectories allow capturing of more subtle changes over time.

Gliomas harboring the isocitrate dehydrogenase (IDH) mutation are molecularly defined as astrocytomas (grades 2-4) or oligodendrogliomas (grades 2 and 3, with 1p/19q codeletion), as per the 2021 WHO classification.^1^ The advent and integration of robust molecular diagnostic markers—most notably IDH1/2 mutation, 1p/19q codeletion, and CDKN2A/B homozygous deletion—has deepened understanding of these tumors’ natural history and response to therapy, guiding both treatment and surveillance decisions.^2^ The rationale for active surveillance in IDH-mutant gliomas lies in their unique biology—characterized by younger age of onset, relatively indolent early clinical behavior and mostly slow tumor kinetics, particularly in low-grade variants. However, despite these favorable features, all IDH-mutant gliomas are diffusely infiltrative tumors that invariably recur or undergo malignant transformation over time with an accelerated growth pattern, and there is no curative treatment. The standard of care for IDH-mutant glioma was historically determined by histologic subtype (astrocytoma vs oligodendroglioma), WHO grade, and patient factors (including age and symptomatology).^3^ Many patients with IDH-mutant gliomas are young adults with concerns regarding cognitive outcomes, fertility preservation, quality of life, and career or family planning.^4^ Upon radiological diagnosis of a presumed low-grade glioma lesion, initial gross total resection, when feasible, can lead to prolonged disease control, but microscopic residual disease and the unavoidable recurrence dictate the need for close monitoring.^5^^,^^6^ In low-risk patients, deferral of further treatment to avoid treatment associated delayed toxicities could maximize quality-adjusted life years, provided clinical and radiographic monitoring ensure timely detection of disease evolution. Early radiologic detection of recurrence or transformation enables the implementation of timely treatment allowing preservation of neurological function. Surveillance should however be individualized based on risk factors, treatment history, and patient status, with multidisciplinary input.^7^ For higher risk IDH-mutant gliomas, the standard remains maximal safe resection followed by radiotherapy and adjuvant chemotherapy with PCV or temozolomide.^8-12^ With the recent positive results of the INDIGO trial, the armamentarium of available therapeutics now includes targeted therapy with IDH inhibitors.^7^^,^^8^^,^^11^ For grade 2 IDH-mutant gliomas, vorasidenib can be considered a first-line adjuvant option for patients with residual or measurable disease after surgery, especially when radiotherapy and chemotherapy are not preferred.^9^^,^^13^ MRI, complementing clinical assessments, constitutes the backbone of glioma surveillance. It is universally recommended for both low-grade and high-grade IDH-mutant gliomas, although the frequency of MRIs may vary based on a number of factors, including tumor grade, histology, tumor volume, and tumor growth rate and whether a patient has undergone therapy or is on active surveillance only.^1^^,^^14^

In this review, we will summarize the current evidence guiding surveillance strategies in IDH-mutant gliomas in the modern molecular era and within an evolving treatment paradigm incorporating IDH inhibitors. Perspectives from neurosurgery, radiology, neuro-oncology, and radiation oncology will be shared to highlight key challenges and future directions in a multidisciplinary discussion.

## Imaging: The Neurosurgeon’s Perspective

Surgical surveillance management of IDH-mutant gliomas begins with the principle of achieving a maximal safe resection at initial diagnosis. Among all modifiable prognostic factors, complete or near-complete resection is the most consistently associated with prolonged survival in lower grade IDH-mutant gliomas.^5^^,^^6^^,^^15^^,^^16^ A gross total resection is frequently feasible and confers a pronounced benefit, particularly in IDH-mutant astrocytomas.^5^^,^^6^^,^^17^ Contemporary surgical strategies rely on functional mapping, high-precision neuro-navigation, and intraoperative MRI to maximize cytoreduction while preserving eloquent cortical and subcortical networks. The initial surgical decisions may influence outcomes for a decade or longer and not only shapes immediate disease control but also establishes the foundation for all subsequent surveillance and therapeutic strategies.^2^ This long-term perspective underscores why an oncologically meaningful resection, rather than merely reducing the tumor volume, is central to optimizing prognosis and preparing patients for future lines of therapy, including targeted approaches.

The presence of any residual tumor, rather than the percentage of tumor removed, appears to be the most relevant prognostic variable.^6^^,^^16^ Consequently, patients whose residual tumors are amenable to complete resection should be offered repeat surgery prior to initiating adjuvant therapy even in higher grade lesions, provided neurological risks are acceptable. Although supramaximal resection may further improve progression-free survival (PFS) in select cases, its true value remains uncertain due to heterogeneous definitions of tumor boundaries beyond the T2/FLAIR abnormality and potential confounding by tumor location and tumor type.^5^^,^^18^^,^^19^ Moreover, such extended resections are typically feasible only in noneloquent regions, raising the possibility that patient selection reflecting underlying biological influences on outcome rather than purely surgical advantage.

The neurosurgical role in surveillance extends beyond the initial intervention. Recurrence should prompt timely reevaluation of resectability, as delayed surgical decision-making may forfeit the window of safe and beneficial second surgical intervention. Approximately 20% of recurrent lower grade gliomas (LGGs) can undergo a second resection without deterioration in functional status, and resections in eloquent or near-eloquent areas do not carry substantially higher risk than at initial surgery when performed with modern mapping techniques.^20^

Surgical planning in both the primary and recurrent setting requires a refined balance: cytoreduction should be maximized to reduce metabolically active tumor burden, while new neurological morbidity must be avoided to maintain long-term functional stability and eligibility for prolonged oral therapy. This paradigm underscores the need for more precise pre- and intraoperative tools capable of delineating biologically relevant margins.

Against this background, intraoperative optical imaging technologies like confocal laser endomicroscopy (CLE) and Raman spectroscopy are gaining importance. Confocal laser endomicroscopy provides subcellular-resolution visualization of tissue architecture following fluorescein administration. In IDH-mutant gliomas—characterized by modest atypia and diffuse parenchymal infiltration—CLE can identify increased cellular density, nuclear pleomorphism, and microvascular alterations. Crucially, CLE enables dynamic assessment of the transition zone between tumor and infiltrated, yet structurally preserved, parenchyma.^21^ Raman spectroscopy offers complementary, label-free molecular profiling by detecting inelastic light scattering and generating tissue-specific biochemical spectra.^22^^,^^23^ It allows pointwise or map-based interrogation of the resection cavity, frequently revealing infiltrative tumor beyond FLAIR-defined boundaries or intraoperative MRI margins.

Integration of CLE and Raman spectroscopy into existing neuro-navigation and functional mapping frameworks may enhance the neurosurgeon’s capacity to optimize cytoreduction, preserve neurological function, and tailor resection strategies to the biological behavior of IDH-mutant tumors. Intraoperative molecular diagnostics allowing the distinction between astrocytoma IDH mutant and oligodendroglioma IDH mutant 1p/19q codeleted may further guide intraoperative decisions, with clinical data showing a more important impact on outcome of residual disease in astrocytoma IDH mutant.^5^^,^^6^^,^^16^^,^^24^^,^^25^

## Imaging Modalities and the Neuroradiologist’s Perspective

Imaging surveillance in IDH-mutant gliomas presents a nuanced clinical challenge, requiring a careful balance between monitoring their typically indolent yet progressive growth and maintaining vigilance for early signs of malignant transformation. Neuroradiology plays a pivotal role in this context by integrating conventional structural MRI preferably using a standardized imaging protocol with advanced imaging modalities to interpret both morphological and biological changes during long-term follow-up.^26^  This standardized brain tumor imaging protocol incorporates high-resolution, three-dimensional (3D) T1-weighted images with and without contrast, T2-weighted, and T2-FLAIR sequences, supplemented as needed by diffusion-weighted imaging (DWI).^26^

**Figure 1.**
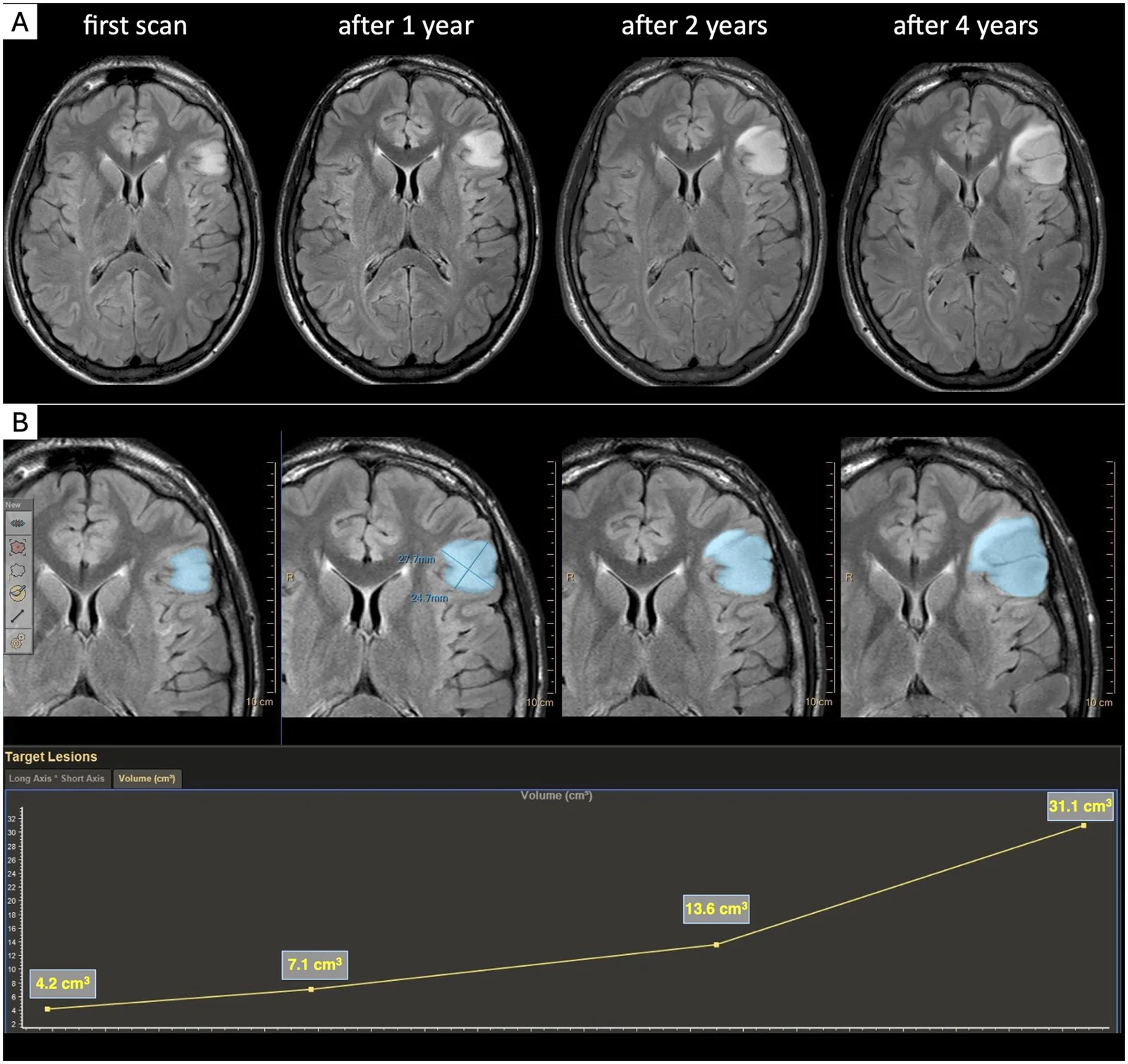
Longitudinal imaging of a left frontal inferior IDH-mutant astrocytoma located in the opercular region, within eloquent language areas, monitored over a 4-year “watch-and-wait” period. (A) Axial FLAIR images qualitatively show progressive and continuous tumor enlargement over time, even in the absence of clinical symptoms. (B) Quantitative volumetric analysis using semiautomatic segmentation on clinical imaging software (Philips IntelliSpace Portal, version 12.1) confirms tumor growth. The volumetric data more precisely illustrates the growth kinetics, revealing a continuous increase in size, with the final tumor volume exceeding 7 times that of the initial scan. Abbreviation: IDH, isocitrate dehydrogenase.

Multiple studies have shown that IDH-mutant gliomas exhibit continuous volumetric expansion, even during clinically stable periods (Figure 1).^27^^,^^28^ This pattern of growth carries significant prognostic weight, with an inverse relationship between the rate of tumor expansion and overall survival.^29^ Specifically, rapid pretreatment growth has emerged as a robust predictor of shortened survival, reinforcing the need for proactive imaging surveillance and timely therapeutic intervention.^27^  Therapeutic options such as chemotherapy, radiotherapy, and targeted IDH inhibitors have demonstrated efficacy in inhibiting tumor growth, making accurate and reproducible imaging follow-up critical for assessing therapeutic response and guiding treatment decisions. The ability to detect subtle changes in tumor dynamics and assess treatment efficacy hinges upon precise measurement methodologies. In this context, the shift from traditional two-dimensional (2D) linear measurements to 3D volumetric quantification represents a pivotal advancement.^30^ The limitations of 2D methods are increasingly recognized, particularly given the infiltrative and irregular geometry of IDH-mutant gliomas. The updated Response Assessment in Neuro-Oncology (RANO) 2.0 criteria reflect this recognition, acknowledging the higher variability and reduced reproducibility inherent in bidimensional measurements.^31^ Such variability can lead to apparent fluctuations in tumor size, which complicate the differentiation between true progression and measurement error.^32^ These inconsistencies risk misinforming clinical decisions, including the premature cessation of effective therapies or unwarranted alterations in treatment strategy.

By contrast, volumetric methods offer a more stable and reliable assessment of tumor burden. Comparative studies demonstrate significantly improved interreader agreement and a substantial reduction in measurement discordance with 3D methods.^32^ Volumetric measures using manual segmentation by experts are unfortunately time-consuming.^33^^,^^34^ Fortunately, semiautomated and automated segmentation tools are emerging and will soon allow faster and reproducible volume assessment in routine practice.^35-37^ Volumetric analysis more accurately reflects tumor growth rates and facilitates early recognition of biologically meaningful changes, which are often precursors to malignant transformation.^28^  While RANO 2.0 retains 2D metrics for widespread accessibility, it formally introduces volumetric assessment as an option.^30^^,^^31^ Regardless of the approach considered, new follow-up images should not be simply compared to the previous images but comparisons must consider longer periods of time. Establishing a growth trajectory over time can help understanding subtle changes in more slowly growing tumors (Figure 1).

During follow-up, awareness of tumor mimics is extremely important. Reactive gliosis after surgery with white matter changes may mimic infiltrative growing tumor on FLAIR/T2 weighted images.^38^ Radiotherapy-associated pseudoprogression in IDH-mutant (IDHmt) tumors typically appears ensue years after the end of radiotherapy often in the shape of waxing and waning frequently periventricular located enhancing lesions, typically smaller in size.^39^^,^^40^ Surgery, radiotherapy, and even chemotherapy have been associated with leuko-encephalopathy mimicking tumor progression.^38^^,^^41^^,^^42^  These can be identified by awareness of the possible occurrence of these developments, by their lack of mass effect and if necessary with more advanced imaging techniques (perfusion MRI, amino acid-positron emission tomography [AA-PET], magnetic resonance spectroscopy [MRS]).

**Figure 2.**
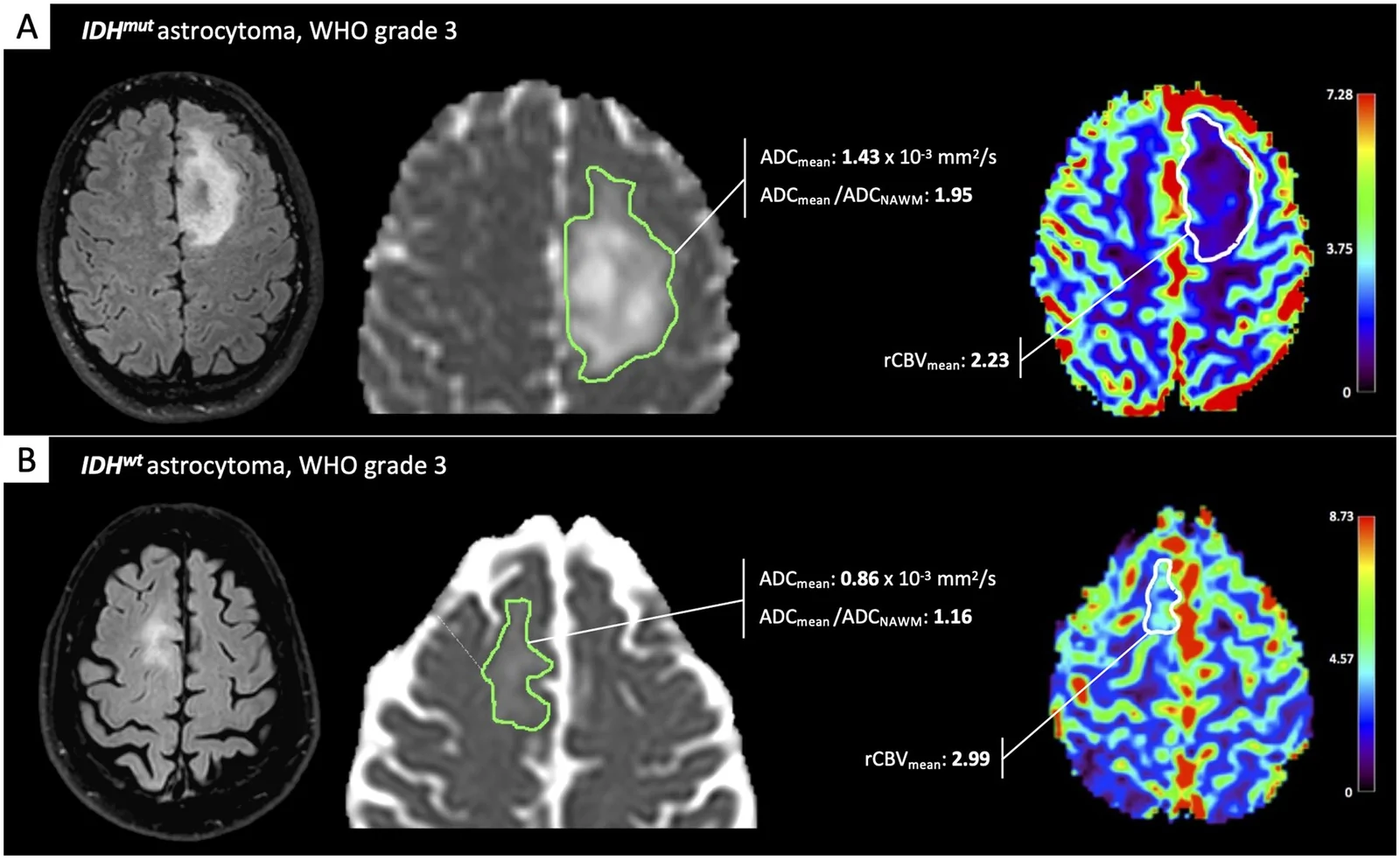
Perfusion and diffusion signatures of IDH mutation in lower grade gliomas. DWI and PWI features from 1 isocitrate dehydrogenase mutant (IDHmut)-LGG (A) and 1 IDH wild type (IDHwt)-LGG (B) were evaluated through a clinically feasible single-slice region of interest (ROI) approach. In contrast to IDH-wildtype LGG, IDH-mutant LGG demonstrates less “aggressive” advanced MRI characteristics, including higher mean ADC/normal-appearing white matter ADC ratio (ADCmean/ADCNAWM) and lower rCBV values. Reprinted from Sanvito et al.,^43^ under the Creative Commons CC BY 4.0 license. Abbreviations: ADC, apparent diffusion coefficient; DWI, diffusion-weighted imaging; LGG, lower grade glioma; PWI, lower perfusion MRI; rCBV, relative cerebral blood volume.

Indeed, contemporary neuroimaging strategies extend beyond measuring changes in tumor size to encompass a wide array of advanced modalities capable of characterizing the biological and metabolic underpinnings of IDH-mutant glioma.^43^^,^^44^ At diagnosis, these tumors typically appear less aggressive than their IDH wild-type counterparts, with a tendency for frontal lobe localization, reduced contrast enhancement, sharper margins, higher apparent diffusion coefficient (ADC) values from DWI and lower perfusion MRI (PWI) parameters (Figure 2).^45-47^ In nonenhancing LGG, the T2-FLAIR mismatch sign is a highly specific and reliable imaging marker for IDH-mutant astrocytoma.^48^ During follow-up, DWI and PWI are key techniques that can capture microstructural and vascular changes that may precede visible growth. The ADC, derived from DWI, serves as an established inverse marker of cellularity, with lower values correlating with increased tumor cell density and potentially heralding malignant progression.^44^^,^^49^ Diffusion tensor imaging (DTI), a related modality, offers additional sensitivity in detecting early microstructural changes in infiltrative tumor regions, particularly under chemotherapy, often preceding alterations visible on standard FLAIR sequences.^50^ Perfusion MRI complements this approach by evaluating vascular parameters such as relative cerebral blood volume (rCBV), a surrogate for angiogenesis.^44^ Longitudinal increases in rCBV, as measured by dynamic susceptibility contrast-PWI, have been shown to anticipate malignant transformation in low-grade gliomas.^51^ More recently, arterial spin labeling (ASL), a noncontrast technique, has gained attention due to its safety profile and potential for long-term use in surveillance. Arterial spin labeling-derived perfusion increases have been detected up to 12 months prior to contrast enhancement on T1-weighted imaging, offering an extended window for therapeutic consideration.^52^ However, the clinical integration of ASL remains limited due to standardization challenges.

Magnetic resonance spectroscopy provides a metabolic lens for glioma evaluation, particularly through the detection of the oncometabolite 2-hydroxyglutarate (2-HG), which is specific to IDH-mutant gliomas. Magnetic resonance spectroscopy-based 2-HG quantification offers both high sensitivity (95%) and specificity (91%) for IDH mutation status, and serves as a direct pharmacodynamic biomarker.^53^^,^^54^ Levels of 2-HG rise with progression and decline in response to effective treatment, thereby enabling real-time, noninvasive monitoring of therapeutic efficacy.^55^^,^^56^ However, clinical adoption is constrained by the technical demands of acquisition and postprocessing, with no standard automated workflow currently available. While largely confined to research settings, 2-HG MRS is poised to play an increasing role in molecular surveillance, particularly in trials involving IDH inhibitors.^57^ Despite the value of advanced MRI techniques, their adoption remains heterogeneous across centers, driven by institutional resources and variations in expertise.^44^^,^^57^^,^^58^ Another advanced MRI technique offering novel insights into tumor metabolism and molecular characteristics is amide-proton transfer-weighted (APTw) imaging.^59^ This chemical exchange saturation transfer method detects endogenous amide protons in mobile proteins and peptides, generating molecular-level contrast. Isocitrate dehydrogenase-mutant LGG generally show lower APTw signal intensity compared to IDH-wildtype lesions.^60^^,^^61^ By quantifying protein content without gadolinium, APT imaging holds promise not only for diagnosis but also for longitudinal surveillance of glioma progression and treatment response.

Molecular imaging further expands the diagnostic armamentarium through AA-PET, utilizing radiotracers such as [18F]FET, [11C]MET, and [18F]fluorodopa. These tracers are actively transported into tumor cells independent of blood-brain barrier integrity, allowing for sensitive detection of tumor metabolism even in nonenhancing gliomas. Positron emission tomography RANO 1.0 criteria now formalize the role of AA-PET in treatment response assessment, basing evaluations on 3D volumetric analysis of metabolically active tumor regions.^62^ This standard further underscores the neuro-oncology field’s transition toward volumetric and biologically grounded metrics. Although currently available primarily in specialized centers, AA-PET offers potential advantages in the surveillance of IDH-mutant gliomas especially when targeted therapies such as IDH inhibitors are used, with a recent study demonstrating earlier detection of metabolic partial response by PET relative to morphological stability on MRI.^63^^,^^64^

Key questions remain in surveillance strategies for IDH-mutant gliomas. A significant consideration is the desire to reduce patient exposure to gadolinium-based contrast agents (GBCAs), particularly in a population that undergoes frequent, long-term surveillance.^65^ Recent data suggest that reliance on contrast enhancement may be reduced without compromising diagnostic integrity. In 1 study, 96.3% of glioma progressions were detectable via T2/FLAIR abnormalities alone, raising questions about the necessity of routine GBCA use in every follow-up scan, especially among long-term survivors.^66^ These findings support emerging guidelines advocating for reduced contrast use during surveillance.^67^

Artificial intelligence (AI) and radiomics are poised to reshape glioma surveillance by enhancing efficiency and standardization. Artificial intelligence-assisted segmentation enhances the reproducibility and efficiency of volumetric assessment, reducing interobserver variability and supporting the integration of automated volumetry into routine clinical software.^27^^,^^35^^,^^37^ Furthermore, AI models are increasingly capable of generating synthetic postcontrast images from noncontrast data, offering a potential alternative to GBCA-based imaging.^65^

In summary, the evolving paradigm of IDH-mutant glioma surveillance is grounded in the integration of quantitative volumetric analysis, biologically informative advanced imaging, and AI-based computational tools. This convergence supports the routine incorporation of these modalities into clinical protocols, positioning neuroimaging not solely as a diagnostic measure but as a dynamic biomarker of disease behavior.

## Systemic Therapy Guiding Surveillance: The Neuro-Oncologist’s Perspective

The slow but continuous growth of lower grade diffuse IDH-mutant gliomas makes active surveillance a key phase of management before introducing further oncological therapy. To determine how best to manage this surveillance period, it is essential to clarify the aims of such strategy. The rationale for active follow-up relies on 3 major objectives. First, active surveillance aims to detect tumor progression at an early stage, enabling timely initiation of new oncological treatment(s). Second, it allows the assessment of short- and long-term tolerance of previous therapies. Third, it provides a framework for a longitudinal and systematic assessment of patients’ needs in terms of supportive care. The goal of this comprehensive approach is to preserve patients’ quality of life for as long as possible.^68^

Early tumor progression detection requires the ability to identify subtle changes in tumor volume. It is now well established that IDH-mutant diffuse grade 2 gliomas follow a slow, initially linear growth pattern.^69^ This growth dynamic persists after tumor resection, with residual tissue progressing at approximately the same rate as preoperatively.^70^ To avoid delayed recognition of tumor growth, 2 key principles must be emphasized. First, it is essential to use the appropriate baseline MRI, meaning the MRI exam obtained at the beginning of active surveillance rather than the most recent pretreatment exam.^71^^,^^72^ Second, with increasing technology allowing (semi)automated tumor segmentation and volumetric assessment, there is a trend toward volumetric evaluation of tumor growth rather than conventional 2D measurements, particularly after surgery, where tumors are frequently reshaped and fragmented, making bidimensional evaluation more of a challenge.^30^^,^^73-76^

Once volumetry is available, however, the cutoff for defining radiological progression remains an issue. While RANO criteria propose a  ≥ 40% increase of tumor volume from baseline as a threshold, this cutoff is intended for clinical trials and may be insufficiently sensitive for use in everyday clinical practice.^31^^,^^32^^,^^76^ Also, the size of the initial lesion and the related absolute increase to call progression in everyday clinical practice are relevant here. Future approaches may require redefining thresholds or identifying the point of inflection of the growth trajectory. This may in turn better capture progression but would also require sufficient and validated specificity.

Importantly, radiological changes must be integrated with systematic clinical evaluation when considering treatment. Epilepsy represents a key clinical marker: seizure evolution may reflect tumor progression but also represents a therapeutic objective in itself, as oncological treatments (surgery, IDH inhibitors, radiotherapy, or chemotherapy) can improve seizure control in pharmaco-resistant cases. Likewise, both subjective cognitive complaints and objective cognitive impairment must be monitored. Indeed, cognitive deterioration (when not explained by other factors) may justify an earlier treatment. Also, in some cases, the anticipated neurological impact of continued tumor growth (ie, the risk of bilateral and cognitive impairment in tumors crossing the corpus callosum) may lead to earlier therapeutic intervention.

Monitoring should also include the longitudinal and systematic evaluation of short- and long-term treatment tolerance in daily practice. Ongoing clinical trials have adopted functional and cognitive primary endpoints, such as survival without neurocognitive deterioration (NCT04702581, NCT02444000) and qualified overall survival integrating functional and cognitive measures (NCT05331521). Similar principles should guide real-world patient management as well, as the goal is not merely to prolong radiographic PFS but to delay cognitive and quality-of-life deterioration, outcomes that matter most to patients and which should guide treatment decisions. Such evaluations are feasible and should be integrated in patients’ care not only during active treatments but also during the active surveillance period.^77^^,^^78^ Patients with IDH-mutant gliomas, even well beyond active oncologic treatment, often continue to experience clinically relevant symptoms that substantially diminish their quality of life.^79^ In this context, the management of patients in active surveillance must include symptom management—particularly epilepsy, cognitive complaints, and psychological distress—together with assistance with maintaining or returning to work and support in terms of social needs.

## Radiation Therapy Guiding Surveillance: The Radiation Oncologist’s Perspective

Decisions about if and when to instigate cytotoxic treatment as an alternative to surveillance are predicated on the likely risk benefit ratio for individual patients. This is especially challenging when considering radiotherapy in this young patient group who are very likely to survive beyond the expected onset of delayed toxicity. Radio-chemotherapy is conventionally reserved for patients considered to present with “high-risk disease,” that is, with a risk on poor outcome without further treatment thus mandating early postoperative treatment. However, a definition of high-risk disease is challenging. Conventional indications of risk using age, tumor size, and laterality are based on studies carried out before current molecular pathology-based integrated diagnosis.^1^^,^^80^^,^^81^ Although tumor genomics are likely a more accurate predictor of risk than clinical factors, grading in the current WHO classification of CNS tumors is still mainly dependent on classical histopathology. Indeed, data emerging from more recent studies reveal sets of molecular markers that may improve patient prognostication.^82^

The evolving understanding of the biological basis of delayed toxicities has led to use of novel radiotherapy technology and other approaches to reflect this, and is also likely to influence risk-benefit in a highly individual manner. Photon RT is typically used to treat these patients and has been shown to be effective in delaying progression and improving survival alongside chemotherapy.^83-85^ Standard doses are 50.4-54 Gy in 1.8 Gy fractions delivered using highly conformal, image guided techniques.^86^^,^^87^ Despite accurate MRI-delineated tumor targeting nontarget brain is inevitably exposed to doses high enough to cause neuronal and vascular toxicity associated with impairment of neurocognitive function (NCF) in the long term.^88-90^ The typical timing and magnitude of this impact remains unclear due to a relative paucity of data and risk of bias inherent in the published literature. However, it is clear that these long-term toxicities may occur as early as 2 years after treatment, are irreversible and potentially have very broad impacts on work and caring responsibilities.^90^^,^^91^ In addition, even small deficits in neurocognition may negatively impact on health-related quality of life and affect activities of daily living.^92^. Other long-term toxicities include endocrine dysfunction due to pituitary damage, skin changes and, changes in hair texture and thickness.

Ongoing studies are attempting to evaluate whether more highly focused radiotherapy, particularly proton beam treatment may spare some of these toxicities since it exposes less nontarget brain to significant doses. Three randomized trials are currently in progress. The phase III APPROACH trial in the United Kingdom will randomize 246 patients with IDH-mutant and 1p19q codeleted tumors that are deemed to require radio-chemotherapy to photons or protons with adjuvant PCV chemotherapy with a primary endpoint of neurocognitive function measured using the EORTC core clinical trial battery composite (CTB COMP).^93^ Additional research will address whether specific brain regions are associated with specific neurocognitive deficits using lesion-symptom mapping. The phase II NRG-BN005 in the United States will include 120 participants treated using PBT or photon RT followed by adjuvant temozolomide chemotherapy (NCT03180502), the primary endpoint is NCF at 10 years, also measured using CTB COMP, as in APPROACH. GliProPh is a German trial of 80 participants with a primary endpoint of neurocognitive function at 3 years (German Clinical Trials Register DRKS00015160). These studies will answer an important question about whether advanced radiotherapy can mitigate concerns about long-term toxicity but will take many years to report data. In the interim, the decision to move from surveillance to radio-chemotherapy should be based on individualized risk-benefit considerations and should involve a multidisciplinary team who can adequately assess the pros and cons of different approaches and convey these to the patient. For patients postradiotherapy, important aspects of follow-up include monitoring for neurocognitive decline and ensuring access to appropriate support and advice if these problems develop.

## Follow-up Interval Recommendations

NCCN and EANO guidelines have proposed expert-opinion-based follow-up schedules for patients with IDH-mutant glioma adapted to the risk of progression, taking tumor type, grade, and prior treatment into account.^3^^,^^11^^,^^94^ More intense follow-up schedules may allow earlier identification of tumor progression, but with unclear impact on overall outcome and which will increase health-care costs and likely to increase anxiety (“scan-anxiety”).^95^ A risk-adapted follow-up schedule considers the time to progression, with shorter follow-up intervals in patients with a shorter expected PFS.^96^ This leads to the recommendation of shorter intervals in case of a “watch-and-wait” policy, after single modality radiotherapy or chemotherapy, in case of larger tumors or with an established higher growth rate and in astrocytoma. Table 1 summarizes PFS as reported in the updates of prospective trials on adjuvant postsurgical treatment that reported on molecular glioma subtypes. Follow-up data and the specified PFS on larger cohorts of patients on a “watch-and-wait” policy are scarce and are usually limited to patients with grade 2 tumors as guidelines recommend immediate postsurgical treatment in grade 3 tumors. One series on 110 predominantly grade 2 oligodendroglioma patients described a median PFS of 69 months, while a similar series on 98 patients with predominantly grade 2 astrocytoma noted a PFS of 4 years.^97^^,^^98^ In a recent large study on grade 2 IDH-mutant glioma, PFS after resection only (*n* = 728) was associated with both extent of resection and tumor type.^5^ The PFS at 5 years for patients with a complete resection was reported as 52% (95% CI, 45-59), as 32% (95% CI, 24-40) for patients with a near total resection and as 82.8% (76-88) for patients with a supramaximal resection.

**Table 1.** Progression-free survival (95% CI) in large prospective trials on (post hoc) molecularly defined grades 2, 3 IDH-mutant (IDHmt) glioma, and as reported in 2 manuscripts on a “watch-and-wait” policy.

Tumor	Trial	Treatment	*n*	Median PFS (yrs)	10 year PFS (%)
Astrocytoma IDHmt grade 2					
	RTOG 9802^84^	RT/PCV	18	10.4	
	EORTC 22033	TMZ	84	3.0 (2.4-3.6)	
		RT	94	4.0 (3.4-5.0)	
Astrocytoma IDHmt grade 3	CATNON10^10^	RT plus adj TMZ	113	6.5 (4.6, 10.4)	
	NOA4^99^	PCV/TMZ	36	3.1	
Oligodendroglioma grade 2					
	RTOG 9802^84^	RT/PCV	18	NR	≈80
	EORTC 22033	TMZ	60	5.4 (3.4-6.6)	
		RT	49	4.6 (3.7-6.9)	
Oligodendroglioma grade 3					
	EORTC 26051^12^	RT/PCV	43	13.1 (5.7-18.6)	56.7 (40.3-70.1)
	RTOG 9402^12^	RT/PCV	58	9.8 (4.5-16.2)	49.7 (36.4-63.1)
	NOA4	PCV/TMZ	33	7.5	

The EANO 2021 glioma guideline suggests initial imaging intervals of 2-6 months upon completion of therapy while noting that longer intervals might be appropriate in cases of durable disease control and less aggressive tumors.^3^ The NCCN guidelines version 2.2025 offer more granularity, and recommend a higher follow-up frequency for patients (1) with grade 3 tumors, (2) with astrocytoma, (3) followed in a watch-and-wait strategy, and (4) treated with either single modality radiotherapy or chemotherapy (Table 2).^94^ The recent SNO-ASCO guideline did not provide follow-up interval recommendations.^11^ Importantly, the follow-up of patients can have legal ramifications, and adhering to national guidelines or institutional policies may therefore be prudent.

**Table 2.** Guidelines for follow-up of isocitrate dehydrogenase-mutant (IDHmt) glioma (adapted from National Comprehensive Cancer Network guidelines version 2.2025).

Grade 2 lesions: First follow-up scan 4 months after surgery	
Oligodendroglioma, IDH mutated, 1p/19q codeleted	After RT and chemotherapy: At least every 6-9 months until progression After RT or chemotherapy: Every 3-4 months in the first 5 years, consider thereafter not longer than 6 months intervals until progression After surgery only: Every 3-4 months until tumor progression. For patients who underwent gross total resection: consider after 1 year every 6 months until tumor progression On vorasidenib: first scan after 4 months, after 1-2 years every 6 months in stable or responding patients also depending on prevorasidenib tumor growth rate if available
Astrocytoma, IDH mutated	After RT and chemotherapy: At least every 6 months until progression After RT or chemotherapy: Every 3-4 months in the first 5 years, consider thereafter not longer than 6 months until progression After surgery only: Every 3-4 months until tumor progression, for patients who underwent gross total resection consider after 2 years every 6 months On vorasidenib: first scan after 4 months, after 1-2 years every 6 months in stable or responding patients also depending on prevorasidenib tumor growth rate if available
Grade 3 lesions: First post radiotherapy scan 4 months after the completion of radiotherapy	
Oligodendroglioma, IDH mutated, 1p/19q codeleted	After RT and chemotherapy: At least every 6-9 months until progression After RT or chemotherapy: Every 3-4 months in the first 5 years, thereafter as frequently as every 3-4 months, but not longer than 6 months until progression
Astrocytoma, IDH mutated	After RT and chemotherapy: At least every 6 months until progression After RT or chemotherapy: Every 3-4 months until progression

An immediate postoperative scan (within 48-72 h from surgery) in IDH-mutant grades 2 and 3 tumors is important to define the extent of resection and should include diffusion weighted images to identify zones of postsurgical ischemia. However, postsurgical changes can also lead to overestimation of the amount of residual tumor.^100-102^ A subsequent MR scan after 3-4 months will allow better delineation of the residual tumor volume, and will inform if unsuspected rapid growth is present. In virtually all grade 2 IDH-mutant glioma patients, decisions for further postsurgical treatments can be postponed until that moment. It is noteworthy that after surgery reactive changes may develop over time confined to the white matter that may mimic tumor recurrence.^38^ For patients treated with radiotherapy, an MR scan shortly after the end of radiotherapy in grades 2 and 3 IDH-mutant glioma carries mainly the risk of picking up pseudoprogression as tumor progression at that stage is very unlikely. Therefore, it is justified to make a first baseline MR scan 4 months after the completion of radiotherapy in patients with IDH mutated grades 2 and 3 tumors, and then continue with scans every 6 months. Watch-and-wait strategies without prior histological verification of presumed low-grade tumors based on neuro-imaging only carry the risk of underestimating the grade of malignancy and thus require initial intervals of only 2-3 months between scans. This is especially true for patients over 50 years of age as the risk of a glioblastoma IDHwt with low-grade histological features presenting with a nonenhancing tumor increases with age.^3^^,^^103^ These intervals can be made longer in case of demonstrated radiological stability after several follow-up scans. In the setting of “watch-and-wait” following surgery only, the NCCN guidelines recommend to have intervals of 3-4 months. A low growth rate is expected in most patients with untreated grade 2 IDHmt glioma with long progression-free intervals especially in patients having undergone a very extensive resection or in patients with oligodendroglioma IDHmt 1p/19q codeleted. Expanding this interval to every 6 months in case of no or only minimal changes after an initial period with follow-up scans every 4 months allowing the establishment of the tumor growth rate is to be considered. Importantly, any new sign or symptom or an increase in seizure frequency not otherwise explained should lead to the consideration of an earlier MRI. This also holds true when radiological changes occur that may be therapy related. In that case, more frequent follow-up will be necessary until lesions stabilize and/or resolve, to ensure tumor progression is not missed.

There is still little clinical follow-up data to guide the management of patients treated after surgery with an IDH inhibitor alone. It seems prudent to follow the recommendations for patients on a “watch-and-wait” policy, until more information becomes available. With a first scan after 4 months and after 1-2 years increasing the intervals to 6 months in stable or responding patients seems reasonable. If patients have a known and slow tumor growth rate prior to the start of vorasidenib, longer intervals can be implemented earlier.

In a subset of patients, in particular those with grade 2 and even grade 3 oligodendroglioma, long-term PFS (anaplastic oligodendroglioma: 30% PFS at 20 years) is observed after radiotherapy in combination with chemotherapy.^12^^,^^84^ In that situation, annual follow-up could be discussed with the patient.

## Conclusions

Surveillance of patients with IDH-mutant glioma is a critical phase in the neuro-oncologic care pathway. It must encompass both early detection of tumor progression and timely initiation of the next therapy, as well as continuous support for patients facing daily challenges related to the disease and the short- and long-term side effects of prior treatments.^104^ The imaging frequency should reflect the risk of tumor progression in the individual patient and should depend on the postresection tumor residue, the postsurgical adjuvant treatment, the established growth rate if known, and the histological diagnosis and tumor grade. Good quality imaging with a standardized imaging protocol is required, but the role of routine gadolinium administration during follow-up of nonenhancing grades 2 and 3 IDH-mutant glioma could be evaluated in prospective series. Routine 3D volumetric analysis may allow a more robust analyses of the tumor size than 2D assessment, but requires routine access to DICOM segmentation and analyses tools. The role of advanced MRI modalities (such as MR perfusion, MR spectroscopy for 2HG, DTI, and volumetric analysis) as well as PET using amino acid tracers ([18F]FET, [18F]FDOPA) remain to be established in the routine surveillance paradigm for IDH-mutant gliomas. Changes in the clinical or radiological status of patients need to be discussed in a multidisciplinary tumor board, to ensure the perspective of all involved medical disciplines. Indeed, Anno 2025 a multidisciplinary approach is critical for the optimal care of these patients, with open multidisciplinary discussions throughout the disease trajectory of these patients to ensure all options and perspectives are considered.
